# Evaluating causal associations of chronotype with pregnancy and perinatal outcomes and its interactions with insomnia and sleep duration: a mendelian randomization study

**DOI:** 10.1186/s12884-024-07023-8

**Published:** 2024-12-18

**Authors:** Qian Yang, Maria C. Magnus, Fanny Kilpi, Gillian Santorelli, Ana Goncalves Soares, Jane West, Per Magnus, Siri E. Håberg, Kate Tilling, Deborah A Lawlor, M Carolina Borges, Eleanor Sanderson

**Affiliations:** 1 MRC Integrative Epidemiology Unit, University of Bristol, Bristol, UK; 2 Population Health Sciences, Bristol Medical School, University of Bristol, Bristol, UK; 3 Centre for Fertility and Health, Norwegian Institute of Public Health, Oslo, Norway; 4 Bradford Institute for Health Research, Bradford Teaching Hospitals NHS Foundation Trust, Bradford, UK; 5 National Institute for Health Research Bristol Biomedical Centre, University Hospitals Bristol NHS Foundation Trust and University of Bristol, Bristol, UK; 6 Office room OF28, Oakfield House, Oakfield Grove, Clifton, Bristol, BS8 2BN UK

**Keywords:** Chronotype, Sleep, Pregnancy and perinatal outcomes, Mendelian randomization

## Abstract

**Background:**

Observational studies suggested chronotype was associated with pregnancy and perinatal outcomes. Whether these associations are causal is unclear. Our aims are to use Mendelian randomization (MR) to explore (1) associations of evening preference with stillbirth, miscarriage, gestational diabetes, hypertensive disorders of pregnancy, perinatal depression, preterm birth and offspring birthweight; and (2) differences in associations of insomnia and sleep duration with those outcomes between chronotype preferences.

**Methods:**

We conducted two-sample MR using 105 genetic variants reported in a genome-wide association study (*N* = 248,100) to instrument for lifelong predisposition to evening- versus morning-preference. We generated variant-outcome associations in European ancestry women from UK Biobank (UKB, *N* = 176,897), Avon Longitudinal Study of Parents and Children (ALSPAC, *N* = 6826), Born in Bradford (BiB, *N* = 2940) and the Norwegian Mother, Father and Child Cohort Study (MoBa, *N* = 57,430), and extracted equivalent associations from FinnGen (*N* = 190,879). We used inverse variance weighted (IVW) as main analysis, with weighted median and MR-Egger as sensitivity analyses. Relying on the individual participant data from UKB, ALSPAC, BiB and MoBa, we also conducted IVW analyses of insomnia and sleep duration on the pregnancy and perinatal outcomes, stratified by genetically predicted chronotypes.

**Results:**

In IVW and sensitivity analyses, we did not find robust evidence of associations of chronotype with the outcomes. Insomnia was associated with a higher risk of preterm birth among evening preference women (odds ratio 1.61, 95% confidence interval: 1.17, 2.21), but not among morning preference women (odds ratio 0.87, 95% confidence interval: 0.64, 1.18), with an interaction P-value = 0.01. There was no evidence of interactions between insomnia and chronotype on other outcomes, or between sleep duration and chronotype on any outcomes.

**Conclusions:**

This study raises the possibility of a higher risk of preterm birth among women with insomnia who also have an evening preference. Our findings warrant replications due to imprecise estimates.

**Supplementary Information:**

The online version contains supplementary material available at 10.1186/s12884-024-07023-8.

## Background

Hypertensive disorders of pregnancy (HDP) and gestational diabetes are common cardiometabolic complications, affecting ~ 15% pregnancies [1]. Together with preterm birth (PTB), these are in turn associated with adverse perinatal mortality and morbidity [2, 3]. Whilst stillbirth is rare in high income countries [4], evidence suggests that 15.3% of pregnancies result in a miscarriage [5], with considerable grief for women and their partners from pregnancy loss at any stage in pregnancy. Systematic reviews of observational studies and recent multivariable regression analyses in large birth cohorts have found insomnia, short (< 6–7 h/day) or long (> 9 h/day) sleep duration was associated with stillbirth [6, 7], gestational diabetes [6, 8, 9], HDP or its subtypes [6, 10], perinatal depression [10, 11], PTB [6, 12], or LBW [10]. Mendelian randomization (MR) is less prone to confounding than observational studies, as genetic variants which are randomly allocated at meiosis are used as instrumental variables (IVs) [13]. Recent MR studies support associations of insomnia with miscarriage, perinatal depression and low offspring birthweight (LBW) [11], shorter sleep duration with perinatal depression, and shorter and longer sleep duration with LBW [10].

Chronotype refers to a person’s circadian preference, defined as morning-, evening- or no-preference. Chronotype is assessed either by self-report or actigraphy-defined timing of when the person is most active [14–20]. Observational studies suggest late sleep midpoint (reflecting evening preference) is associated with an increased risk of gestational diabetes in a US cohort (*N* = 7524) and its subsample of actigraphy data (*N* = 782), but not HDP in the same participants [14, 15]. Late sleep midpoint was not associated with pregnancy loss in another US cohort (*N* = 1088) [16], or small-for-gestational age in a Chinese cohort (*N* = 11,192) [17]. The first US cohort also showed an increased risk of PTB in women with late sleep midpoints (> 5 a.m.) [18], while a Chinese cohort showed the opposite, with an increased risk of PTB among those with early midpoints (< 2.45 a.m.) [17]. A few studies suggested that chronotype was associated with perinatal depression or mood [20–22], while other studies showed null associations [19, 23–25]. These apparent inconsistencies could be due to variation in sample sizes, with all being small (*N* = 26 to 299). To the best of our knowledge, we have found no MR studies of chronotype on pregnancy/perinatal outcomes [26].

Poor sleep quality and unhealthy sleep duration have been observed within certain groups of chronotype preferences in pregnant women [15, 16, 19, 20], and large studies of non-pregnant people [27, 28]. People examining interactions of insomnia and sleep duration with chronotype revealed associations not seen when these sleep traits are studied independently of chronotype in young or middle-aged adults [29].

The aim of this study was to explore associations of a lifetime genetic predisposition to evening- versus morning-preference on stillbirth, miscarriage, gestational diabetes, HDP, perinatal depression, PTB, LBW, macrosomia, and birthweight using two-sample MR. Based on our previous findings of insomnia and sleep duration [10, 11], this study also explored differences in their associations with these outcomes between women with morning- and evening-preference.

## Methods

### Participants

**Fig. 1:**
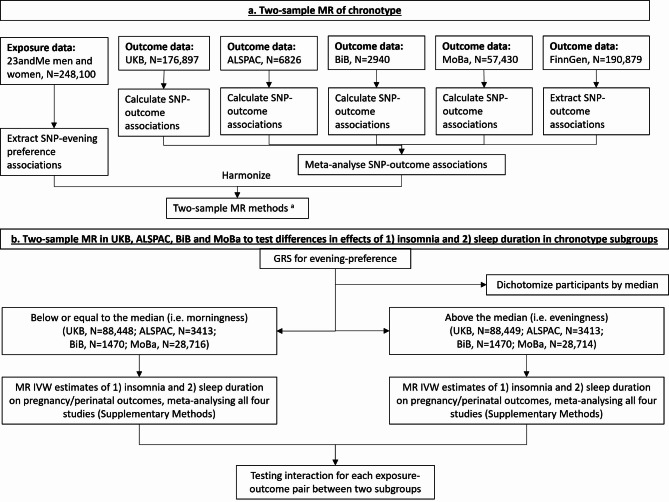
Summary of methods and data contributing to our two-sample MR analyses. ^a^ Two-sample MR methods include IVW, weighted median, MR-Egger and leave-one (SNP)-out analysis. Abbreviations: ALSPAC, Avon Longitudinal Study of Parents and Children; BiB, Born in Bradford; GRS, genetic risk score; IVW, inverse variance weighted; MoBa, Norwegian Mother, Father and Child Cohort Study; MR, Mendelian randomization; SNP, single nucleotide polymorphism; UKB, UK Biobank

This study used data from the MR-PREG collaboration, which aims to explore causes and consequences of different pregnancy and perinatal outcomes [10, 11, 30]. We used individual-level data from UK Biobank (UKB, *N* = 176,897 women who had experienced at least one pregnancy), and mother-offspring pairs from Avon Longitudinal Study of Parents and Children (ALSPAC, *N* = 6826), Born in Bradford (BiB, *N* = 2940) and Norwegian Mother, Father and Child Cohort Study (MoBa, *N* = 57,430), and summary-level data from FinnGen (the nationwide network of Finnish biobank with 173,746 women). FinnGen (R9) is a large repository of summary gene-disease association data and for pregnancy conditions the control groups include women who have never been pregnant [31]. Figure 1 shows how each cohort contributed to our MR analyses. All studies had ethical approval from relevant national or local bodies and participants provided written informed consent. Details of their recruitment and genotyping are described in Additional file 1: Text S1. We followed the ‘Strengthening the Reporting of Observational Studies in Epidemiology using Mendelian randomization’ (STROBE-MR) guidelines to report this study (Additional file 2: STROBE-MR checklist) [32].

### Pregnancy and perinatal outcomes

We included ever experiencing stillbirth, ever experiencing miscarriage, and PTB (gestational age < 37 completed weeks), gestational diabetes, HDP, perinatal depression, LBW (birthweight < 2500 g), macrosomia (birthweight > 4500 g), and birthweight as a continuous measure. Full details about how these outcomes were measured and derived in each participating study and how we harmonised them across studies are in Additional file 3: Table S1. In UKB, gestational age was only available for a subset of women (*N* = 5362) who had a child born during or after 1989, the earliest date for which linked electronic hospital perinatal data were available. Therefore, we have assessed LBW and macrosomia rather than small- and large-for-gestational age for which cases would be too few for precise estimation. If multiple pregnancies were enrolled in the birth cohorts, we randomly selected one pregnancy per woman. In FinnGen, it was only possible to study miscarriage, gestational diabetes, HDP and PTB, where women with pre-existing hypertension were included in HDP cases.

### Chronotype and IVs

The most recent genome-wide association study (GWAS) of chronotype combines data from UKB and 23andMe, though sex-specific results were not available [33]. We extracted summary results of genome-wide significant associations available in 23andMe (without UKB) participants for two-sample MR, to minimize potential bias due to its overlap with our outcome sample [34]. Among the 248,100 23andMe participants included in the most recent GWAS, ≥ 97% of them were of European ancestry, and ~ 44.8% of them were female.

In 23andMe, the question used to measure chronotype (“Are you naturally a night person or a morning person?”) was asked in two surveys [33, 35]. Response options were initially “night owl”, “early bird”, and “neither”, but changed to “night person”, “morning person”, “neither”, “it depends”, and “I’m not sure” [33, 35]. As shown in Additional file 3: Table S2, participants with discordant (morning preference in one survey but evening preference in the other) or neutral (“neither”, “it depends”, and “I’m not sure”) answers to both questions were excluded from the GWAS [33], who accounted for ~ 13% of 23andMe participants [35]. Participants with a neutral and a non-neutral (“night owl”, “early bird”, “night person” and “morning person”) answers were included solely based on the non-neutral one [33]. Thus, the GWAS included 127,622 and 120,478 individuals of evening- and morning-preference, respectively, and identified 110 genome-wide significant (P-value < 5 × 10^− 8^) single nucleotide polymorphisms (SNPs) in autosomes using linear mixed model to account for population structure [33]. We used the ‘clumping’ function from ‘TwoSampleMR’ R package to check if those SNPs were independent [36], and 105 SNPs (Additional file 3: Table S3) were retained based on a threshold of R^2^ < 0.01 using all European samples from the 1000 Genomes Project as the reference population.

### Insomnia, sleep duration and IVs

Information on insomnia and sleep duration was collected at the UKB initial assessment centre, and described in Additional file 1: Text S1. Characteristics of genome-wide significant SNPs for insomnia (81 SNPs) and sleep duration (78 SNPs) were extracted from the largest GWAS available, where linear mixed model were used to account for population structure [37, 38], and listed in Additional file 3: Table S4. None of these SNPs were overlapped with the 105 SNPs for chronotype. Insomnia (r_g_=-0.10, P-value = 0.113) and sleep duration (r_g_=0.11, P-value = 0.142) were genetically weakly correlated with chronotype in UKB women using linkage disequilibrium score regression [39], and their IVs have been used in previous MR studies [10, 11].

### Statistical analyses

#### Two-sample MR of chronotype

The 105 SNP-chronotype associations were from 23andMe GWAS [33]. We generated SNP-outcome associations in UKB, ALSPAC, BiB and MoBa using logistic regression for binary outcomes and linear regression for birthweight, adjusting for genotyping batch (only applicable in UKB and MoBa), women’s age and top 10 principal components. We also extracted female-specific associations of the 105 SNPs with miscarriage, gestational diabetes, HDP and PTB from summary-level data of FinnGen where population structure was controlled via linear mixed model [31], and meta-analysed SNP-outcome associations across five studies using fixed-effects with inverse variance weights.

We used MR inverse variance weighted (IVW) method as main analysis, which meta-analyses each coefficient calculated as the SNP-outcome association divided by the SNP-exposure association with a fixed effect model [40]. We repeated IVW analyses by leaving one cohort out in turn to explore between-study heterogeneity. To exam the MR relevance assumption, the strength of IVs was calculated as the proportion of variance of evening-preference and related mean F-statistic of the 105 SNPs [41]. We further compared sex-combined associations from 23andMe with the equivalent associations in UKB women, whose measurement of chronotype was described in Additional file 1: Text S1. To explore violation of the MR exclusion restriction assumption (i.e. unbalanced horizontal pleiotropy), sensitivity analyses included estimations of between-SNP heterogeneity using Q-statistic and leave-one (SNP)-out analysis, and MR with weighted median and MR-Egger approaches [42, 43]. The association of maternal with fetal genotype for sleep traits might also introduce bias for any outcomes in the index pregnancy, which are plausibly influenced by fetal genotype [44]. To explore this, we used data from MoBa, where trio data were available, to compare SNP-outcome (except for stillbirth and miscarriage history) associations with adjustments for (1) fetal genotypes (*N* = 43,649), (2) both fetal and paternal genotypes (*N* = 28,214), versus (3) without those adjustments (*N* = 57,430).

#### Two-sample MR stratified on genetically predicted chronotype

We compared associations of insomnia and sleep duration with the outcomes between women with a genetically predicted evening- versus morning-preference (Fig. 1). The analyses were undertaken on individual-level data in UKB and birth cohorts. We constructed a weighted genetic risk score of the 105 genome-wide significant SNPs for evening preference using external weights from 23andMe, and separate women into two subgroups using its median (Additional file 1: Fig S1) [45].

Among women below and above the median, we obtained two-sample MR IVW estimates of insomnia and sleep duration for each outcome (Additional file 1: Fig S2). Briefly, in UKB we generated SNP-exposure and SNP-outcome associations in a split cross-over samples design [46]. We also conducted two-sample MR using SNP-exposure associations from UKB, and SNP-outcome associations from birth cohorts, and further meta-analysed MR estimates from all four cohorts using fixed-effects with inverse variance weights (Additional file 1: Text S1) [10, 11]. Differences between results among women below and above the median were calculated using the ratio of odds ratios (ORs, i.e. OR_1_/OR_2_) with its standard error (SE) equalling to $\:\sqrt{{{\text{S}\text{E}}_{1}+\text{S}\text{E}}_{2}}$ [47]. Thus, lower and upper bound of a 95% confidence interval (CI) are exp (log OR_1_ – log OR_2_ ± 1.96 × SE), and an interaction P-value was derived from the z-score (i.e. $\:\frac{\text{l}\text{o}\text{g}\:{\text{O}\text{R}}_{1}-\:\text{l}\text{o}\text{g}\:{\text{O}\text{R}}_{2}}{\sqrt{{{\text{S}\text{E}}_{1}+\text{S}\text{E}}_{2}}}$) referring to the normal distribution. For birthweight, the same approach was taken using the difference in differences in mean between the two subgroups [47].

Two-sample MR analyses were conducted using ‘TwoSampleMR’ R package [36]. All analyses were performed using R 3.6.1 (R Foundation for Statistical Computing, Vienna, Austria).

## Results

Table 1 summarizes characteristics of included women from UKB, ALSPAC, BiB, MoBa and FinnGen. In two-sample MR, the 105 SNPs explained approximately 0.68% of the variance in chronotype (Additional file 3: Table S2), and their mean F-statistic was 16.1 among 248,100 participants. The association of all of these 105 SNPs with evening preference in 176,887 UKB women was directionally consistent, though weaker, than the equivalent using GWAS 23andMe women and men (Additional file 1: Fig S3).

**Table 1:** Characteristics of women from UKB, ALSPAC, BiB, MoBa and FinnGen included in this study

Variable	UKB (*N* = 176,897)	ALSPAC (*N* = 6826)	BiB (*N* = 2940)	MoBa (*N* = 57,430)	FinnGen (*N* = 190,879)
Country	UK	UK	UK	Norway	Finland
Year of recruitment	2006–2010	1991–1992	2007–2010	1999–2009	1986-present
	*Mean (standard deviation)*				
Age at delivery ^a^	25.5 (4.6) years	28.7 (4.7) years	26.8 (6.0) years	30.4 (15.1) years	Not available
Gestational age	38.9 (3.8) weeks	39.6 (1.7) weeks	39.7 (1.9) weeks	39.4 (2.1) weeks	Not available
Offspring birthweight	3186.7 (547.6) grams	3441.5 (523.0) grams	3357.9 (571.2) grams	3600.0 (562.1) grams	Not available
Sleep duration	7.2 (1.1) hours	Not available	Not available	8.0 (1.5) hours ^b^	Not available
	*N (%)*				
Offspring sex, male	Not available	3430 (50.2)	1504 (51.2)	29,315 (51.0)	Not available
Having morning preference	103,190 (58.3)	Not available	Not available	Not available	Not available
Having insomnia	57,318 (32.4)	909 (13.3) ^b^	Not available	Not available	Not available
	*N cases/N controls (prevalence*,* %)*				
Ever experiencing stillbirth	4907/107,791 (4.4)	48/4546 (1.0)	31/2588 (1.2)	255/39,560 (0.6)	Not available
Ever experiencing miscarriage	42,717/107,791 (28.4)	1378/4546 (23.3)	14/2588 (0.5)	10,090/39,560 (20.3)	15,073/135,962 (10.0)
Preterm birth	551/4811 (10.3)	285/4931 (5.5)	172/2706 (6.0)	2879/49,586 (5.5)	8108/135,806 (5.6)
Gestational diabetes	726/170,308 (0.4)	34/6283 (0.5)	136/2657 (4.9)	470/56,524 (0.8)	11,279/179,600 (5.9)
HDP	2128/174,769 (1.2)	1099/5698 (16.2)	347/2159 (13.8)	7300/49,866 (12.8)	13,071/177,808 (6.8)
Perinatal depression	5168/20,860 (19.9)	423/5896 (6.2)	312/2245 (12.2)	2554/49,977 (4.9)	Not available
Low offspring birthweight	13,429/149,084 (8.3)	337/6376 (5.0)	167/2725 (5.8)	1675/52,905 (3.1)	Not available
Macrosomia	2716/149,084 (1.8)	113/6376 (1.7)	42/2725 (1.5)	2468/52,905 (4.5)	Not available

^a^ UKB women were recruited with an average age 56.9 (standard deviation 7.8) years, and FinnGen participants were recruited with an median age of 63 years [31].

^b^ Data were not used in this two-sample MR.

Abbreviations: ALSPAC, Avon Longitudinal Study of Parents and Children; BiB, Born in Bradford; GRS, genetic risk score; HDP, hypertensive disorders of pregnancy; MoBa, the Norwegian Mother, Father and Child Cohort; UKB, UK Biobank

**Fig. 2:**
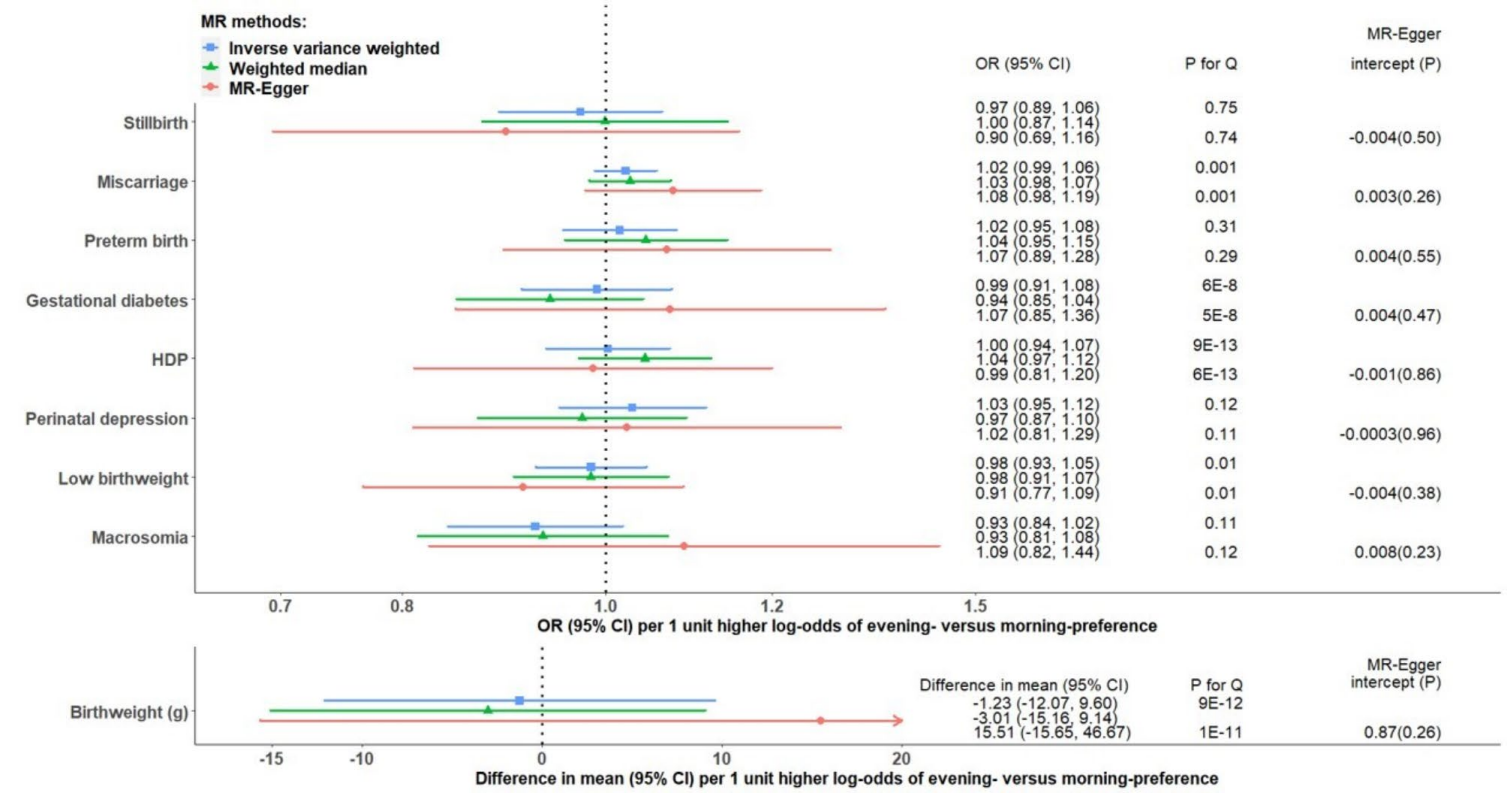
Two-sample MR estimates for causal effects of chronotype on pregnancy and perinatal outcomes meta-analysing UK Biobank, three birth cohorts and FinnGen. P-value for Cochran’s Q-statistic testing statistical evidence for between-SNP heterogeneity in the two-sample MR estimates. P-value for MR-Egger intercept < 0.05 suggests unbalanced horizontal pleiotropy. Abbreviations: CI, confidence interval; HDP, hypertensive disorders of pregnancy; MR, Mendelian randomization; OR, odds ratio

In the main IVW analyses combining the five studies, there was little evidence of associations of chronotype with the outcomes with all 95% confidence intervals (CIs) including the null (Fig. 2). There was no strong statistical evidence of between-study heterogeneity, and the removal of the largest studies (UKB or FinnGen) did not materially change the null effect, though resulted in wider CIs, as would be expected (Additional file 1: Fig S4). Between-SNP heterogeneity was observed for miscarriage, gestational diabetes, HDP, LBW and birthweight (Fig. 2), as a few individual SNPs showed associations with the same outcomes (Additional file 1: Fig S5). However, leave-one (SNP)-out analyses suggested little evidence of a single SNP driving our IVW results (Additional file 1: Fig S6). Furthermore, sensitivity analyses using weighted median and MR-Egger approaches were consistent with the null associations for all outcomes, and the MR-Egger intercept did not provide evidence of unbalanced horizontal pleiotropy for any outcomes (Fig. 2). After adjusting for fetal genotype or both fetal and paternal genotype in MoBa, most SNP-outcome associations were statistically consistent with the unadjusted results (i.e. 95% CI for the difference between two associations including the null), with exceptions for ≤ 2 SNPs with gestational diabetes, HDP, perinatal depression, LBW, macrosomia and birthweight (Additional file 1: Fig S7 and Additional file 3: Table S5).

**Fig. 3:**
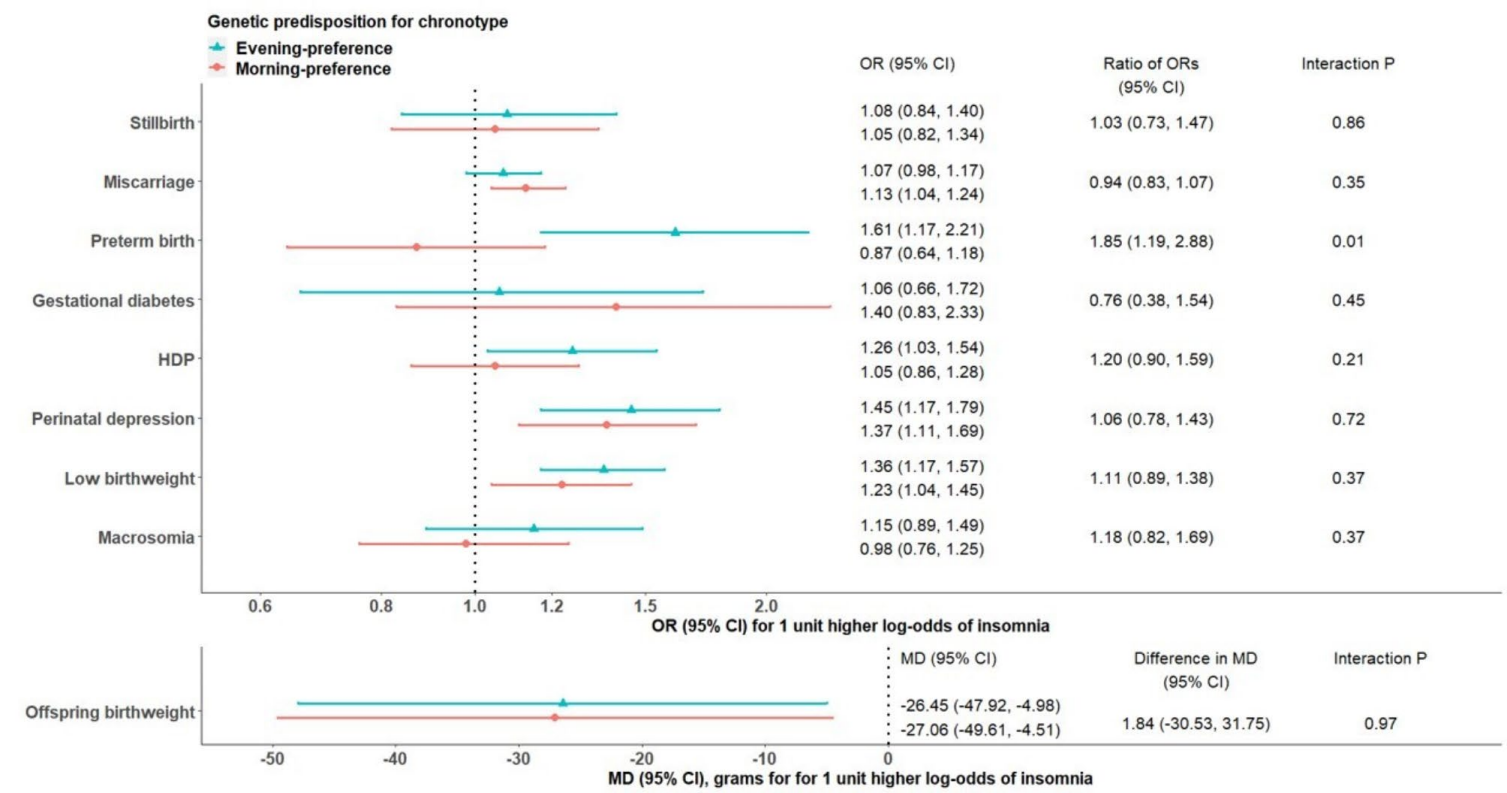
Two-sample MR IVW estimates meta-analysing UK Biobank and three birth cohorts for causal effects of insomnia on pregnancy and perinatal outcomes, stratified on chronotype. Abbreviations: CI, confidence interval; IVW, inverse variance weighted; MD, mean difference; MR, Mendelian randomization; OR, odds ratio

**Fig. 4:**
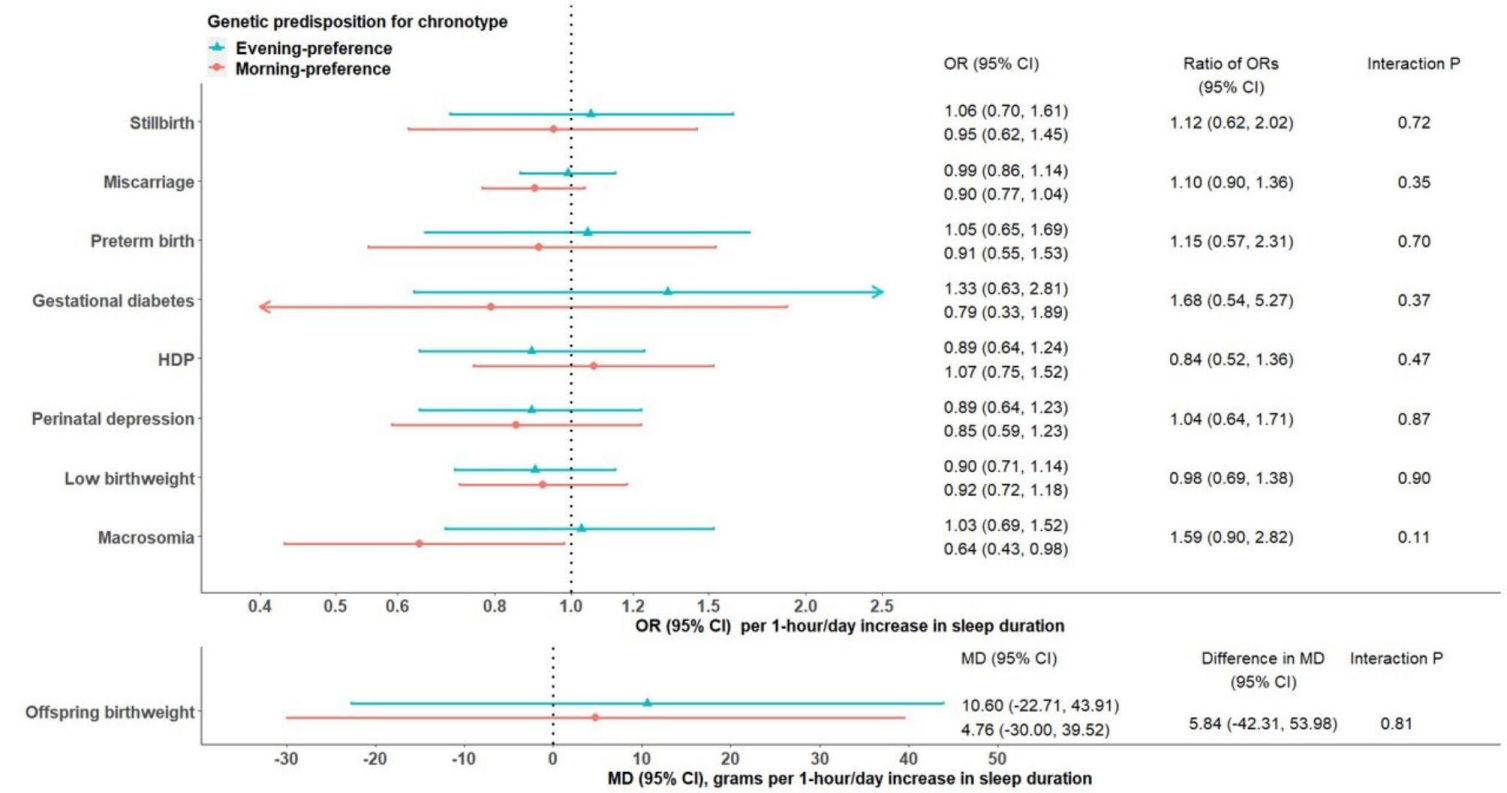
Two-sample MR IVW estimates meta-analysing UK Biobank and three birth cohorts for causal effects of sleep duration on pregnancy and perinatal outcomes, stratified on chronotype. Abbreviations: CI, confidence interval; IVW, inverse variance weighted; MD, mean difference; MR, Mendelian randomization; OR, odds ratio

After combining all individual-level data (i.e. UKB, ALSPAC, BiB and MoBa), insomnia was associated with PTB among women with genetic predisposition to evening preference (OR 1.61, 95% CI: 1.17, 2.21), but not among morning preference women (OR 0.87, 95% CI: 0.64, 1.18). We observed a significant interaction between these two sleep traits on risk of PTB (P-value = 0.02). Associations of insomnia with other outcomes (Fig. 3) and sleep duration with all outcomes (Fig. 4) were similar in morning- versus evening-preference subgroups (interaction P-value > 0.05).

## Discussion

Our study used a novel application of MR to explore associations of maternal chronotype with adverse pregnancy and perinatal outcomes. Based on our previous findings of insomnia and sleep duration [10, 11], we further investigated whether their associations with the outcomes differ between chronotype preferences. We interpret our results as estimating the association of a lifetime genetic predisposition to a trait (e.g. evening- (versus morning-) preference) on the basis that SNPs are determined at conception [48, 49]. In comparison to other sleep related traits, few studies have explored associations of chronotype with pregnancy and perinatal outcomes, with details summarized in Additional file 3: Table S6. Thus our results (particularly the suggestive interaction between insomnia and chronotype for PTB) need further replications.

Our null associations of chronotype with stillbirth history, miscarriage history, HDP and LBW were broadly consistent with previous observational studies of pregnancy loss [16], HDP [14, 15], and small-for-gestational age [17]. Our null association of chronotype with perinatal depression was largely in agreement with two small observational studies of self-report chronotype on depressive symptoms scores from 2nd trimester to 16 weeks postpartum [19, 20]. One exception was at 2 weeks postpartum, when a statistically significant higher score (indicating severer depressive symptoms) was observed among women with evening- (versus morning-) preference [20]. In contrast to our findings, a US prospective cohort study found self-report evening preference was associated with higher risks of gestational diabetes and PTB [14, 18], though a Chinese cohort found morning preference, assessed using accelerometer, was associated with a higher risk of PTB [17]. Differences between these two cohorts, and between them and our MR results could reflect differences in how chronotype was measured, residual confounding, and true population differences. They could also reflect the fact that the observational studies explored associations of chronotype assessed in pregnancy, where as our MR analyses are of genetically predicted lifetime exposure to chronotype possibility with potential horizontal pleiotropy (discussed in limitations).

Consistent with our previous MR study, including all women irrespective of chronotype [11], insomnia was associated with higher risks of miscarriage, perinatal depression, and LBW in both morning- and evening-preference subgroups with little evidence for interactions. We identified an interaction between chronotype and insomnia with PTB, with increased risk of PTB in those with insomnia symptoms and reporting an evening preference, and no evidence of an association in those with morning preference. In our previous study in all women there was no robust evidence that insomnia was associated with PTB [11]. The underlying mechanism is yet to be identified. Possible pathways could include maternal glycaemic profile, blood pressure and inflammation all of which are causally associated with PTB [1, 3, 50], and may be influenced by insomnia or chronotype in general population [51–53]. Why these associations would lead to an interaction, and whether they really do, require further research. Furthermore the interaction we have observed requires replication in independent studies before we can assume it is causal. If our findings are replicated, they could provide a basis for identifying women at risk of PTB early in pregnancy, by based on their report of both insomnia symptoms and an evening preference. Randomized controlled trials exploring the effects of cognitive behavioural therapy on reducing perinatal depression have shown that it reduces insomnia in pregnancy [54–56]. Our findings might support those trials to further explore its effects on PTB particularly in women with evening preference. MR studies to investigate potential interactions between sleep traits and other environment factors that are causally associated with some pregnancy and perinatal outcomes could further inform this area.

### Strengths and limitations

The main strengths of our study included (1) an exploration of multiple pregnancy and perinatal outcomes of clinical importance and public health concern; (2) use of MR to explore associations of a lifetime predisposition to morning or evening preference; (3) exploring whether associations of insomnia and sleep duration with those outcomes interact with chronotype; (4) a number of sensitivity analyses to explore how robust our results were to MR assumptions; and (5) large sample sizes for some important outcomes, e.g. ever having a miscarriage, which have not been explored previously in observational or MR studies.

Our two-sample MR may be vulnerable to weak instrument bias [34], and we were not able to directly test the relevance of genetic IVs for chronotype (i.e. MR relevance assumption) in pregnant people or women at their reproductive ages, as none of our birth cohorts measured chronotype. We showed directionally consistent, though weaker, associations with chronotype in UKB women versus the combined 23andMe women and men. As we used SNPs selected from 23andMe women and men as IVs, if there was a true weaker IV-exposure (chronotype) effect in women, we may have underestimate potential effects of lifetime evening preference on pregnancy and perinatal outcomes. The MR assumption of no confounding between the genetic instrument and outcome might be violated in our analyses due to assortative mating [57], as a weak correlation of chronotype between women and their partners was observed [58]. Population structure was controlled for in our study to mitigate against it causing confounding. Our results may be biased by horizontal pleiotropy resulting in a path from the genetic instrument to the outcomes. For example, some SNPs that we used as instrumental variables for chronotype are known to associate with education attainment, anthropometric measures, and other health related factors [59], which could influence the outcomes explored here. However, none of our sensitivity analyses provided evidence of substantial bias due to unbalanced horizontal pleiotropy. We only applied IVW when testing interactions, as weighted median and MR-Egger approaches provided some wide CIs even before splitting the participants [10, 11]. Thus, those findings require replications using pleiotropy robust MR methods in a larger sample and non-European populations. A monotonicity assumption is required for our MR estimates to be interpreted as a local average treatment effects [13]. This means our genetic IVs for sleep traits (e.g. chronotype) cannot increase the probability of reported exposure (e.g. evening preference) in some women while decrease it in others [60, 61], though it is difficult to evaluate the influence of violating this assumption on MR studies.

We did not apply any corrections for multiple testing, as it is more relevant to a hypothesis-searching study (e.g. GWAS) than to our hypothesis-driven design [48]. We acknowledge that the interaction we observed could be due to chance, and emphasized the importance of further replications with sufficient cases to secure statistical power, and triangulation of evidence across different methods with different sources of bias [62]. Chronotype preference was self-reported via single questions in 23andMe and UKB rather than via standard questionnaires that include multiple categories and items, and this could result in misclassification. Measurement errors in self-report insomnia and sleep duration and potential selection bias in UKB might influence MR results, which have been comprehensively discussed in our previous MR studies [10, 11]. Our study focused on chronotype preference rather than an actigraphy observed sleep midpoint that has been suggested to be a more accurate measure of circadian rhythm [63]. Hence we are careful to interpret our results as relating to a persons reported preference. This measure does have the advantage that it would be easy to capture in antenatal clinics. We are not able to assess the phenomenon known as ‘social jetlag’, where someone’s daily life, such as childcare, work, and other commitments, means that they have to be active at a time (morning or evening) when they would prefer not to be. Our study did not include small- or large-for-gestational age that would be of more clinical importance than LBW and macrosomia. Further MR studies with large number of cases on these outcomes are needed.

## Conclusions

Our findings suggest little evidence that a genetic lifetime predisposition to evening compared to morning preference is associated with pregnancy and perinatal outcomes, but raises the possibility that women with predispositions to an evening preference and insomnia are at a higher risk of PTB. These findings require replications due to their uncertainty as reflected by wide CIs for some outcomes, and exploration in non-European populations.

## Electronic supplementary material

Below is the link to the electronic supplementary material.

Addition file 1: Text S1. Supplementary Methods. **Fig. S1**. Histogram of the GRS for evening preference. **Fig. S2**. Summary of data in two-sample MR stratified on chronotype. **Fig. S3**. SNP-eveningness associations in 23andMe versus UKB. **Fig S4.** Leave-one study-out analyses for two-sample MR of chronotype on outcomes. **Fig S5**. Forest plots of two-sample MR of chronotype on outcomes. **Fig S6**. Leave-one SNP-out analyses for two-sample MR of chronotype on outcomes. **Fig S7**. Comparisons of SNP-outcome associations in MoBa. **Fig S8.** Flow chart.

Additional file 2: STROBE-MR checklist.

Additional file 3: **Table S1**. Definitions of outcomes. **Table S2**. Definition of chronotype in 23 and Me. **Table S3**. List of SNPs for chronotype and their female-specific estimates in UKB. **Table S4**. List of SNPs for insomnia and sleep duration. **Table S5**. Associations of chronotype SNPs with outcomes in MoBa, adjusting for fetal and paternal genotype **Table S6**. Results extracted from previous studies. **Table S7**. Associations of chronotype SNPs with outcomes in UKB, ALSPAC, BiB and MoBa. **Table S8**. Associations of insomnia SNPs with outcomes in UKB, ALSPAC, BiB and MoBa. **Table S9**. Associations of sleep duration SNPs with outcomes in UKB, ALSPAC, BiB and MoBa. **Table S10**. Female-specific estimates of IV-insomnia and IV-sleep duration associations in UKB.

## Data Availability

We used both individual participant cohort data and publicly available summary statistics. We present summary statistics that we generated from those individual participant cohort data in Additional file 3: Tables S7-S10. Full information on how to access UKB data can be found at its website (https://www.ukbiobank.ac.uk/researchers/). All ALSPAC data are available to scientists on request to the ALSPAC Executive via this website (http://www.bristol.ac.uk/alspac/researchers/), which also provides full details and distributions of the ALSPAC study variables. Similarly, data from BiB are available on request to the BiB Executive (https://borninbradford.nhs.uk/research/how-to-access-data/). Data from MoBa are available from the Norwegian Institute of Public Health after application to the MoBa Scientific Management Group (see its website https://www.fhi.no/en/op/data-access-from-health-registries-health-studies-and-biobanks/data-access/applying-for-access-to-data/ for details). Summary statistics from FinnGen are publicly available on its website (https://finngen.gitbook.io/documentation/data-download).

## Abbreviations

ALSPAC: Avon Longitudinal Study of Parents and Children
BiB: Born in Bradford
CI: confidence interval
GWAS: Genome-wide association study
HDP: hypertensive disorders of pregnancy
IVs: instrumental variables
IVW: inverse variance weighted
LBW: low offspring birthweight
MoBa: Norwegian Mother, Father and Child Cohort Study
MR: Mendelian randomization
PTB: preterm birth
OR: odds ratio
SE: standard error
SNPs: single nucleotide polymorphisms
STROBE-MR: Strengthening the Reporting of Observational Studies in Epidemiology using Mendelian randomization. UKB: UK Biobank
